# Transcriptomic Identification of Diagnostic Biomarkers for Alcohol-Associated Liver Cirrhosis: Integration of Population-Level Epidemiology with Multi-Cohort Transcriptomic Analysis

**DOI:** 10.3390/ijms27135809

**Published:** 2026-06-26

**Authors:** Hao Wang, Wenzhang Ding, Linjie Zhang, Muyang Xu, Jing Sui

**Affiliations:** 1 School of Electronic and Information Engineering, Anhui Jianzhu University, Heifei 230601, China; wh518812@stu.ahjzu.edu.cn (H.W.); myxu@stu.ahjzu.stu.cn (M.X.); 2 School of Clinical Medicine, Wannan Medical University, Wuhu 241002, China; 24101010298@stu.wnmc.edu.cn; 3 School of Public Administration, Nanjing University of Information Science and Technology, Nanjing 210044, China; zhanglinjieedu@163.com; 4 School of Public Health, Southeast University, Nanjing 210009, China

**Keywords:** alcohol-associated liver cirrhosis, drug repurposing, diagnostic biomarkers, hepatic fibrosis, Immune microenvironment, machine learning, weighted gene co-expression network analysis

## Abstract

Alcohol-associated liver cirrhosis (ALC) lacks aetiology-specific molecular diagnostic biomarkers. This study aims to quantify the association between alcohol and cirrhosis risk, and to identify transcriptomic diagnostic biomarkers and candidate therapeutics. Methods: Survey-weighted logistic regression was applied to 17,007 adults from NHANES (2017–2023) to quantify alcohol-cirrhosis associations. ALC transcriptomic data from four GEO datasets were analysed using weighted gene co-expression network analysis (WGCNA) and three parallel machine learning algorithms (LASSO, Random Forest, SVM-RFE). External validation was performed in an independent cohort of 93 samples. Candidate therapeutics were identified via drug signature database querying and validated by molecular docking. Heavy drinking conferred a 5.14-fold increased cirrhosis risk (95% CI: 2.60–10.20, *p* < 0.001). Transcriptomic analysis revealed global downregulation of long non-coding RNAs (with 91.7% of dysregulated lncRNAs being suppressed). A five-gene diagnostic signature (*IL1B*, *CCL3*, *LUM*, *SPP1*, *ITGA6*), specifically developed to distinguish ALC from histologically normal liver tissue, achieved an area under the receiver operating characteristic curve (AUC) of 0.824 in an external validation cohort. Immune infiltration analysis uncovered global contraction of macrophage-associated transcriptomic signatures across M0, M1, and M2 subtypes, inversely correlated with fibrotic hub gene upregulation. Fluvastatin and honokiol were identified as candidate therapeutic agents, with strong binding affinities to IL1B and CCL3, respectively. This study confirms a dose-dependent alcohol-cirrhosis association and establishes a five-gene diagnostic signature (distinguishing ALC from normal liver tissue) alongside candidate therapeutics, warranting prospective clinical validation. The identified tissue-derived signature and therapeutic candidates provide a foundation for future ALC-specific diagnostic and therapeutic strategies; their translation into a non-invasive (e.g., blood-based) assay will require dedicated validation in circulating samples.

## 1. Introduction

Alcohol-associated liver cirrhosis (ALC) represents the end-stage of alcohol-related liver disease, contributing disproportionately to global cirrhosis-related mortality [1,2]. Unlike other aetiologies such as viral hepatitis or metabolic dysfunction-associated steatotic liver disease (MASLD), ALC is driven by direct ethanol toxicity and gut-derived endotoxemia [3]. Despite its profound clinical burden, therapeutic options to reverse established fibrosis remain severely limited, and current diagnosis relies heavily on invasive liver biopsy or non-specific clinical indices [4]. Consequently, validated, ALC-specific molecular biomarkers—and ultimately the non-invasive assays that could be derived from them—are urgently needed. Non-invasive diagnostic strategies for ALC remain inadequate; while transient elastography and serum fibrosis indices are widely used, none are ALC-specific, and their accuracy in distinguishing ALC-related fibrosis from other aetiologies remains limited [5].

Recent advances in high-throughput sequencing highlight the regulatory roles of long non-coding RNAs (lncRNAs) and mRNAs in hepatic fibrogenesis [6]. In ALC specifically, lncRNAs have been shown to regulate hepatocyte injury, lipid metabolism, inflammation, and hepatic stellate cell (HSC) activation through complex interactions with miRNAs and mRNAs [6,7]; however, their precise regulatory networks in end-stage alcoholic cirrhosis remain poorly characterised. Yet, in ALC specifically, the contribution of lncRNA-mediated regulatory networks to disease-specific fibrotic and immune programmes remains poorly characterised, partly because most transcriptomic studies have focused on protein-coding genes alone. While WGCNA and machine learning algorithms have successfully identified biomarkers in other liver diseases [8,9], and non-invasive diagnostic tools for ALD have advanced considerably in recent years [5], a comprehensive ALC-specific lncRNA-mRNA co-expression network analysis combined with multi-algorithm feature selection remains lacking. Furthermore, lncRNAs have emerged as key regulators of hepatocyte injury, inflammation, and fibrogenesis in ALD [6,7], yet their precise regulatory networks in end-stage alcoholic cirrhosis—particularly in concert with immune remodelling—remain poorly characterised.

A recent dose–response meta-analysis confirmed that alcohol consumption accelerates cirrhosis morbidity and mortality in a dose-dependent manner, with mortality risk accelerating at higher consumption levels [10]. To address this critical gap, we employed a two-stage integrative framework. First, we quantified the population-level association between heavy alcohol consumption and cirrhosis risk using data from 17,007 adults in the National Health and Nutrition Examination Survey (NHANES). Second, we integrated human ALC transcriptomic datasets to construct co-expression networks, applied a multi-algorithm ML approach (LASSO, Random Forest, and SVM-RFE) to isolate a robust diagnostic gene signature, and validated it in an independent external cohort. Finally, computational drug repurposing and molecular docking were conducted to nominate novel therapeutic candidates. Together, this framework is designed to yield a tissue-based diagnostic signature and mechanistically grounded therapeutic leads for ALC, providing a rational basis for subsequent non-invasive assay development.

## 2. Results

### 2.1. Baseline Characteristics and the Epidemiological Link Between Alcohol and Cirrhosis

Among the 17,007 NHANES participants, 80 were identified with liver cirrhosis. As summarised in Table 1, patients with cirrhosis were significantly older (58.81 vs. 48.56 years, *p* < 0.001) and had a higher prevalence of diabetes and smoking (both *p* < 0.001). Clinically, the cirrhosis cohort exhibited markedly worse hepatic profiles, including elevated AST, ALT, and FIB-4 index, alongside reduced platelet counts (all *p* < 0.001). Notably, the proportion of heavy drinkers was more than three-fold higher in the cirrhosis group than in the non-cirrhosis group (49.8% vs. 14.7%, *p* < 0.001).

To determine whether these group differences reflected an independent association, multivariable, survey-weighted logistic regression was performed (Table 2). In the fully adjusted model (Model 3), which accounted for demographics, education, BMI, and diabetes, a history of heavy drinking independently conferred a 5.14-fold increased risk of cirrhosis (95% CI: 2.60–10.20, *p* < 0.001). Furthermore, a significant dose–response relationship was observed: each additional daily alcoholic drink increased the risk of cirrhosis by 18% (OR = 1.18, 95% CI: 1.07–1.30, *p* = 0.002). These population-level findings establish a robust epidemiological foundation for the subsequent molecular analyses.

### 2.2. Transcriptomic Landscape and Global lncRNA Suppression

**Figure 1:**
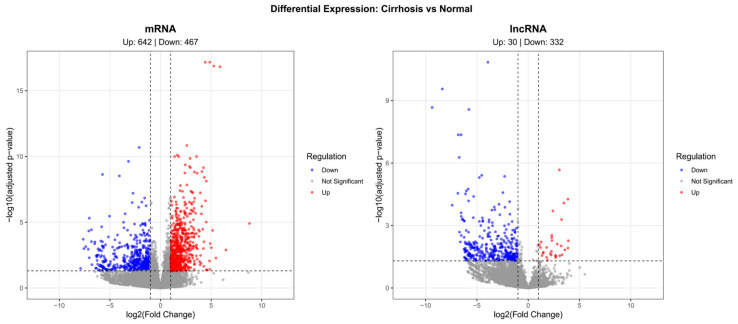
Differential expression of mRNAs and lncRNAs in alcoholic liver cirrhosis. Volcano plots of differentially expressed genes between ALC (*n* = 6) and normal liver tissues (*n* = 12). (**A**) mRNA: 642 upregulated (red), 467 downregulated (blue). (**B**) lncRNA: 30 upregulated (red), 332 downregulated (blue). Grey: not significant. Dashed lines: adjusted *p*-value = 0.05 and |log2FC| = 1.

**Figure 2:**
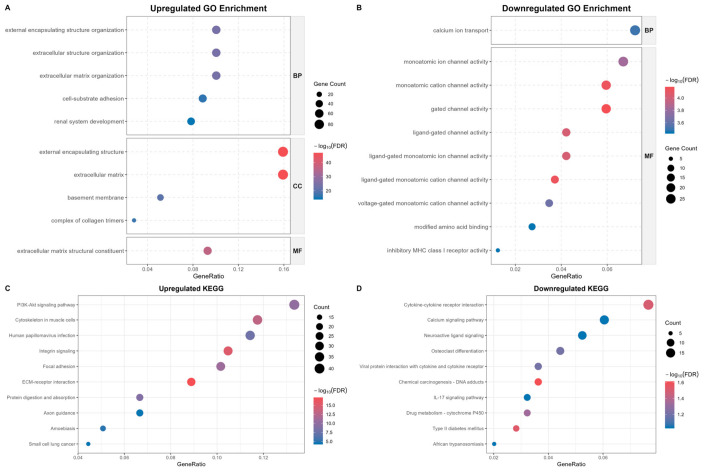
Functional enrichment analysis of DEGs. (**A**,**B**) Top 10 GO terms for upregulated (**A**) and downregulated (**B**) genes, faceted by biological process (BP), cellular component (CC), and molecular function (MF). (**C**,**D**) Top 10 KEGG pathways for upregulated (**C**) and downregulated (**D**) genes. Pathways were ranked by adjusted *p*-value (FDR), with only the globally most significant 10 terms shown per direction to enhance visual clarity. Dot size represents gene count, and colour represents –log_10_(FDR).

Differential expression analysis of ALC tissues identified 1471 differentially expressed genes (DEGs; adjusted *p* < 0.05 and |log2FC| > 1) (Figure 1). While protein-coding mRNAs exhibited a relatively balanced pattern of dysregulation, long non-coding RNAs (lncRNAs) displayed a profoundly asymmetric pattern: 332 were significantly downregulated versus only 30 upregulated. This pronounced asymmetry (91.7% downregulated) suggests that global lncRNA silencing may represent a candidate molecular feature of ALC pathophysiology, warranting further investigation. Functional enrichment revealed that upregulated DEGs were predominantly involved in extracellular matrix (ECM) organisation and PI3K-Akt signalling, whereas downregulated genes were enriched in immune-related signalling, ion homeostasis, and xenobiotic metabolism pathways (Figure 2). To reduce visual complexity, only the top 10 most significant pathways (ranked by adjusted *p*-value) are displayed for each comparison. 

### 2.3. Weighted Gene Co-Expression and Interaction Networks

**Figure 3:**
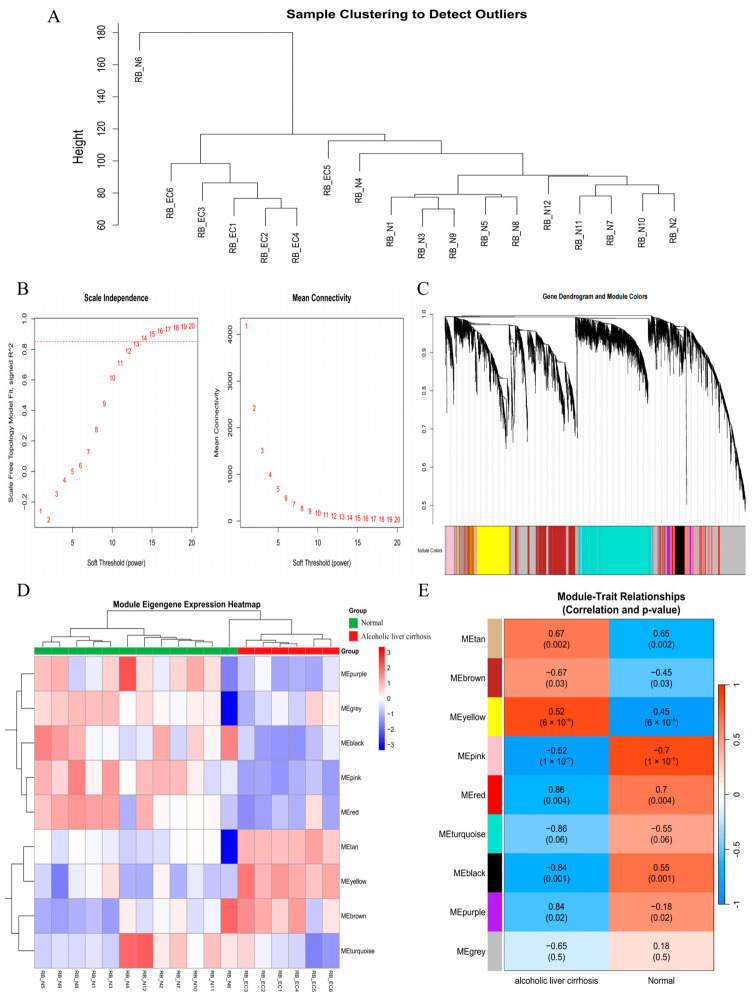
Construction of the weighted gene co-expression network. (**A**) Sample clustering dendrogram. (**B**) Scale-free topology fit index (signed R^2^, left) and mean connectivity (right) across candidate soft-thresholding powers. In the left panel, each number denotes a candidate soft-thresholding power, and the red dashed line marks the scale-free topology fit threshold (signed R^2^ = 0.85); the smallest power reaching this threshold (β = 14) was selected for network construction. (**C**) Gene clustering dendrogram with 9 identified modules. (**D**) Module eigengene expression heatmap. (**E**) Module–trait relationship heatmap.

**Figure 4:**
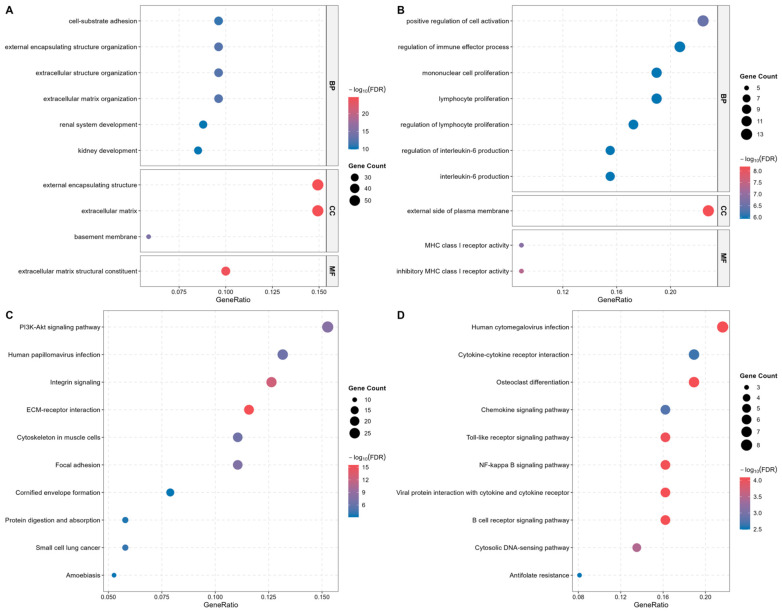
Functional enrichment analysis of the Yellow and Pink modules. (**A**,**C**) Top 10 GO terms (**A**) and KEGG pathways (**C**) for the Yellow module, which was enriched in fibrosis-related functions. (**B**,**D**) Top 10 GO terms (**B**) and KEGG pathways (**D**) for the Pink module, which was enriched in immune-related functions. Pathways were ranked by adjusted *p*-value (FDR), with only the 10 most significant terms shown per module to reduce complexity. Dot size reflects gene count; colour gradient represents –log_10_(FDR).

To capture system-level properties, WGCNA was applied, identifying nine distinct co-expression modules (Figure 3). Module-trait correlation analysis highlighted the “Pink” module as having the strongest negative correlation with ALC, while the “Yellow” module showed the most significant positive correlation. Consistent with overall ALC pathology, the Yellow module was heavily enriched in fibrogenesis and ECM–receptor interactions, whereas the Pink module was enriched in immune signalling pathways (Figure 4). The most significantly enriched terms (top 10 by adjusted *p*-value) for each module are shown in the figure. The gene composition of all nine modules is summarised in Supplementary Table S1.

**Figure 5:**
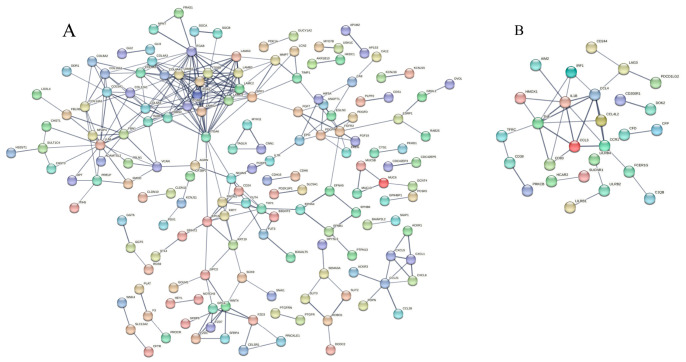
PPI networks of the Pink and Yellow modules. (**A**) Pink module: 57 nodes, 34 edges. (**B**) Yellow module: 389 nodes, 310 edges. Networks constructed using STRING database (interaction score ≥ 0.700).

Comprehensive lncRNA-mRNA co-expression and pathway interaction networks for the non-key modules are provided in Supplementary Figures S1 and S2. For the critical ALC-associated modules, the Pink network centred around the downregulated lncRNA TMEM26-AS1, linking to inflammatory mediators, whereas the Yellow network was orchestrated by the upregulated lncRNA UCA1, driving fibrotic pathways (Supplementary Figure S3). Subsequent protein–protein interaction (PPI) network construction prioritised core regulatory nodes corresponding to these modules (Figure 5).

### 2.4. Machine Learning-Based Diagnostic Signature Identification

To derive a candidate diagnostic panel from these hub genes, three parallel machine learning algorithms—LASSO regression, Random Forest, and SVM-RFE—were applied. The detailed feature selection processes and algorithmic metrics for the Pink and Yellow modules are systematically documented in Supplementary Figures S4 and S5, respectively. The intersection of these algorithms identified a five-gene core signature: *IL1B*, *CCL3*, *LUM*, *SPP1*, and *ITGA6*. A diagnostic model combining these five features achieved a corrected Area Under the Curve (AUC) of 0.922 in the training cohort when evaluated via leave-one-out cross-validation (LOOCV) (Supplementary Figure S6). Given the limited training sample size (*n* = 18), the training AUC (0.922) should be interpreted cautiously; the externally validated AUC of 0.824 in 93 independent samples is considered the primary performance estimate.

### 2.5. CTD-Based Validation of Ethanol–Gene Interactions

To confirm that the five-gene diagnostic signature is biologically responsive to ethanol exposure, we systematically queried the Comparative Toxicogenomics Database (CTD). Direct ethanol–gene interaction evidence was identified for four of the five genes, each supported by distinct peer-reviewed studies. Specifically, CTD records show that ethanol exposure upregulates *IL1B* [11,12,13,14,15] and *CCL3* [14,16,17] and increases SPP1 [18,19] and *ITGA6* [20]. For *LUM*, the CTD does not record a direct ethanol–gene interaction. Instead, an indirect association was identified: combined exposure to carbon tetrachloride and ethanol has been reported to upregulate *LUM* mRNA expression [21]. While this indirect evidence aligns with *LUM’s* established role in alcohol-driven fibrogenesis, it should be interpreted with caution, as the co-exposure context precludes the attribution of this transcriptional alteration to ethanol alone. Collectively, direct ethanol-modulated expression evidence was identified for four of the five signature genes (*IL1B*, *CCL3*, *SPP1*, *ITGA6*), with *LUM* supported by indirect co-exposure data. This nuanced pattern reinforces the biological plausibility of our diagnostic signature while maintaining stringency regarding chemical–gene causal interactions.

### 2.6. External Validation and Characterisation of Macrophage-Associated Transcriptomic Signatures

**Figure 6:**
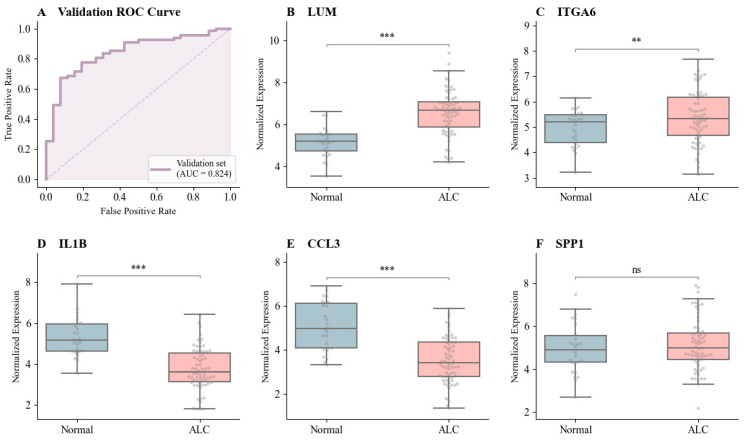
External validation of the diagnostic model and core genes in an independent cohort (*n* = 93). (**A**) ROC curve of the 5-gene diagnostic model in the external validation cohort. (**B**–**F**) Boxplots displaying the expression levels of core genes (*LUM*, *ITGA6*, *IL1B*, *CCL3*, *SPP1*) in normal (blue) vs. ALC (pink) tissues. Statistical significance was determined by Wilcoxon rank-sum test (*** *p* < 0.001, ** *p* < 0.01, ns: not significant). AUC = 0.824.

The diagnostic performance of the five-gene signature was further confirmed in the independent validation cohort, with individual gene expression trajectories remaining consistent across all three contributing datasets (Figure 6).

**Figure 7:**
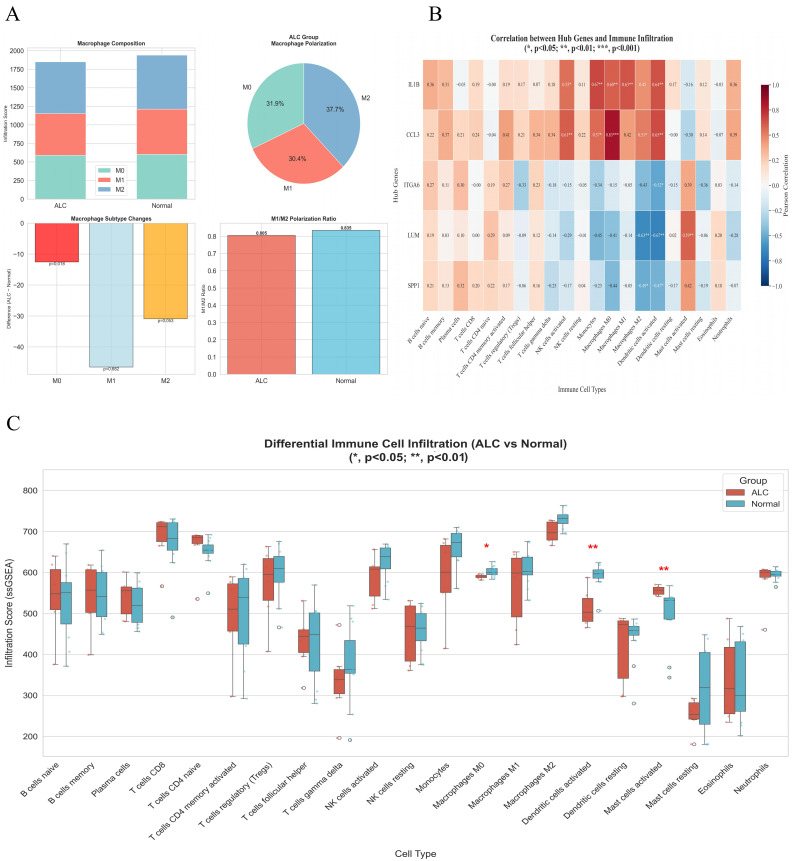
Immune infiltration landscape and hub gene–immune correlations in ALC. (**A**) Macrophage dynamics showing global attenuation of macrophage-associated transcriptomic signatures and M1/M2/M0 distribution. (**B**) Hub gene–immune correlation heatmap for inflammatory hubs (*IL1B*, *CCL3*) and fibrotic hubs (*LUM*, *SPP1*, *ITGA6*). (**C**) Differential infiltration via ssGSEA scores.

To unravel the immunological context of these alterations, ssGSEA was performed. Strikingly, ALC tissues exhibited a systemic contraction of the macrophage pool—including M0, M1, and M2 subtypes. This attenuation of macrophage-associated transcriptomic signatures was inversely correlated with the upregulation of fibrotic hub genes (*LUM*, *SPP1*, *ITGA6*) (Figure 7). This co-occurrence is consistent with—but does not by itself demonstrate—a model in which extensive ECM deposition accompanies a reduced representation of macrophage-associated transcripts. Whether this reflects physical exclusion of immune effectors or an immunosuppressive niche remains a hypothesis to be tested by spatially resolved or single-cell approaches.

### 2.7. Computational Drug Repurposing and Molecular Docking

**Figure 8:**
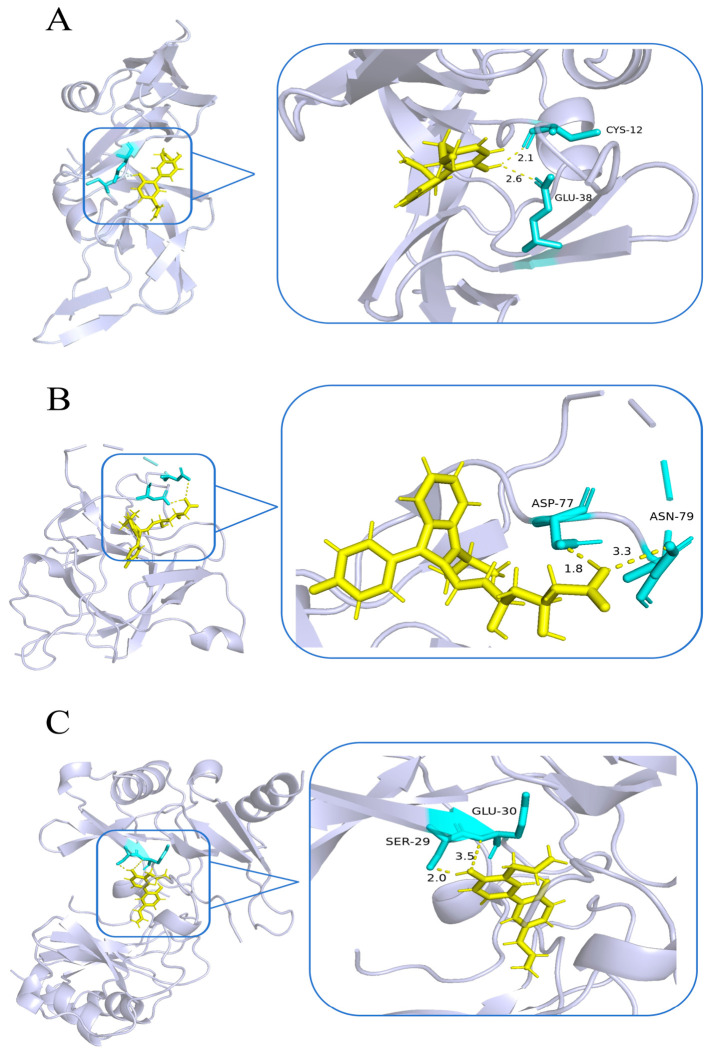
Molecular docking analysis of candidate compounds with target proteins. Three-dimensional visualisation of optimal binding poses. The protein backbone is shown in cartoon representation (light purple), ligands in yellow sticks, and key residues in cyan. Hydrogen bonds are indicated by yellow dashed lines, with distances in angstroms. (**A**) Honokiol-CCL3 (3FPR): hydrogen bonds with CYS-12 (2.1 Å) and GLU-38 (2.6 Å). (**B**) Fluvastatin-IL1B (3POK): hydrogen bonds with ASP-77 (1.8 Å) and ASN-79 (3.3 Å). (**C**) Honokiol-CCL3 (4MHE): hydrogen bonds with SER-29 (2.0 Å) and GLU-30 (3.5 Å).

Leveraging the 5-gene signature, a query of the DSigDB identified several compounds predicted to reverse the ALC-associated transcriptomic pathology, with fluvastatin and honokiol emerging as top candidates. The top-ranked compounds identified from DSigDB querying are summarised in Supplementary Table S2. Molecular docking simulations confirmed stable binding interactions, with fluvastatin achieving a binding affinity of −7.65 kcal/mol to IL1B and honokiol of −6.94 kcal/mol to CCL3 (Figure 8). Specifically, fluvastatin selectively engaged the active domain of IL1B through critical hydrogen bonds, while honokiol demonstrated preferential binding to CCL3.

## 3. Discussion

This integrative study advances the molecular understanding of ALC through two complementary lines of evidence. At the population level, heavy alcohol consumption conferred a 5.14-fold independent risk of cirrhosis in a nationally representative cohort, reinforcing the need for aetiology-specific diagnostic tools [22]. We acknowledge that the qualitative association between heavy drinking and cirrhosis is itself well established. The contribution of the present epidemiological component is therefore not the association per se, but (i) its quantification of a dose–response relationship in a recent, nationally representative U.S. cohort (NHANES 2017–2023), and (ii) its role as the population-level anchor that motivates, and is mechanistically linked—via CTD ethanol–gene evidence—to, the transcriptomic signature. The novelty of this work lies in the integration of population epidemiology, lncRNA–mRNA network analysis, multi-algorithm machine learning and computational drug repurposing within a single framework, rather than in any individual component. At the molecular level, transcriptomic profiling and multi-algorithm machine learning converged on a five-gene signature with an external validation AUC of 0.824, alongside two candidate therapeutics with favourable in silico binding profiles. It is important to note that the drug repurposing strategy employed here operates at the level of transcriptomic signature reversal rather than individual gene inhibition; accordingly, the molecular docking analyses of fluvastatin and honokiol against IL1B and CCL3 reflect target engagement capacity and are interpreted in the context of the candidates’ broader pleiotropic mechanisms, as discussed below.

A salient finding is the pronounced global downregulation of lncRNAs in ALC, with 91.7% exhibiting decreased expression. This contrasts sharply with the balanced distribution among mRNAs and suggests that widespread lncRNA suppression may constitute a candidate molecular feature of end-stage alcoholic liver disease requiring replication in larger independent cohorts. One plausible, though unproven, contributor is epigenetic silencing, as ethanol and acetaldehyde can perturb one-carbon metabolism and chromatin-modifying enzyme activity [23]; loss of functional hepatocyte mass in cirrhosis may further contribute, given the tissue-specific expression of many lncRNAs. We did not test these mechanisms directly and present them only as hypotheses to be examined in future epigenomic studies.

The Yellow and Pink modules provide mechanistic insight into the dual pathological processes driving ALC. The Yellow module was enriched for ECM organisation and integrin signalling, consistent with hepatic stellate cell activation in fibrogenesis [24]. Within this module, lncRNA *UCA1* emerged as a central hub connected to pro-fibrotic genes *SPP1* and *LAMA2*. UCA1 has been implicated in HCC progression [25,26]. Within the limits of a co-expression analysis, our data position it as a hub correlated with pro-fibrotic genes, but the proposed microRNA-sponging mechanism is inferred from other contexts and was not assessed here. Conversely, the Pink module demonstrated enrichment for immune processes, including B cell receptor and NF-κB signalling. The hub lncRNA *TMEM26-AS1* exhibited connectivity to inflammatory mediators *IL1B* and *CCL3*, as well as the ferroptosis pathway. The suppression of this module suggests immune dysfunction rather than simple inflammation, contributing to impaired hepatic immune surveillance in advanced disease [27]. Clinically, this distinction implies that immunostimulatory rather than immunosuppressive strategies may be more appropriate in the end-stage cirrhotic setting.

The five core diagnostic genes bridge the inflammatory and fibrotic arms of ALC pathogenesis. *IL1B* and *CCL3* are well-characterised inflammatory mediators [28,29]. More relevant to the present study, both transcripts are consistently down-regulated in ALC tissues across our training and validation cohorts—a seemingly paradoxical pattern that we interpret below in the context of CAID. Their suppression in end-stage cirrhosis is biologically counterintuitive, yet aligns with emerging concepts of disease-stage-dependent immune remodelling. Our results specifically reflect the transcriptomic landscape of end-stage cirrhosis, a state increasingly recognised as Cirrhosis-Associated Immune Dysfunction (CAID), characterised by progressive immunodeficiency and systemic inflammation that may culminate in functional immune exhaustion [27]. This stands in contrast to early-stage alcohol-associated liver disease, particularly alcoholic hepatitis, which is characterised by a ‘cytokine storm’ and hyperinflammation [30]. Consequently, the suppression of *IL1B* and *CCL3* likely results from two converging mechanisms: the functional exhaustion of innate immune cells described in CAID [27], and the ‘burn-out’ effect caused by massive fibrotic replacement. Histologically, the accumulation of Type I collagen physically displaces functional hepatic parenchyma and resident immune niches [31,32], leading to a dilution of cytokine-producing cells within the dense fibrotic scar tissue. The fibrosis-related genes LUM, SPP1 and ITGA6 are established contributors to ECM remodelling and hepatic fibrosis [33,34]. Most relevant to our findings, SPP1 has a reported stage-dependent, pro-fibrogenic role in advanced fibrosis, consistent with our observation that SPP1 up-regulation maps to the fibrotic Yellow module. Our immune-infiltration analysis revealed an unexpected attenuation of macrophage-associated transcriptomic signatures rather than M2-dominant polarisation. We interpret this correlative observation cautiously: it is compatible with ECM-associated remodelling of the intrahepatic immune compartment, but a causal claim of physical immune-cell exclusion would require spatially resolved or single-cell data, which were not available here. We therefore present it as a hypothesis rather than an established mechanism.

The drug repurposing analysis nominated fluvastatin and honokiol as therapeutic candidates. Fluvastatin demonstrated strong binding affinity to IL1B (−7.65 kcal/mol). Beyond cholesterol-lowering effects, statins possess pleiotropic anti-inflammatory and anti-fibrotic properties through inhibition of RhoA and Rho-kinase signalling [35,36]. Recent clinical evidence demonstrates that statin use is associated with reduced cirrhosis progression and HCC risk in chronic liver disease patients [35,36]. Honokiol exhibited preferential binding to CCL3 (−6.94 kcal/mol) and has demonstrated hepatoprotective effects. Recent experimental studies confirm that honokiol can inhibit epithelial-mesenchymal transition (EMT) and hepatic fibrosis by suppressing the Wnt/β-catenin and TGF-β signalling pathways [37]. It warrants emphasis that the DSigDB-based repurposing approach identifies compounds capable of reversing the disease-associated transcriptomic signature as a whole, rather than exclusively targeting individual downregulated genes. Accordingly, the therapeutic relevance of fluvastatin and honokiol in the end-stage cirrhotic context is more plausibly attributable to their well-documented target-independent mechanisms—RhoA/Rho-kinase inhibition and TGF-β pathway suppression, respectively—than to direct modulation of IL1B or CCL3, which are suppressed at this disease stage. Prospective validation should therefore prioritise earlier fibro-inflammatory stages of ALC, where IL1B and CCL3 are overexpressed and direct anti-inflammatory targeting remains mechanistically coherent. These compounds, identified through unbiased screening with established safety profiles, represent attractive candidates for stage-stratified evaluation. An important question is whether fluvastatin and honokiol modulate the expression or function of the signature genes. Mechanistically, the DSigDB query selects compounds predicted to reverse the disease-associated transcriptomic signature as a whole rather than to inhibit any single gene; the docking results for IL1B and CCL3 (Figure 8) therefore index target-engagement capacity rather than the primary mode of action. To examine the question more directly, we performed a structured literature survey of reported drug–gene and drug–pathway interactions for both candidates against each signature gene. For the fibrotic arm, fluvastatin specifically attenuates hepatic stellate-cell activation and lowers α-SMA, NF-κB and extracellular-matrix gene expression in the liver [38], consistent with the broader anti-fibrotic, RhoA/Rho-kinase-dependent actions of statins [35,36]; honokiol suppresses TGF-β1/SMAD signalling and so attenuates the fibrotic ECM programme encompassing LUM, SPP1 and ITGA6 [39]. For the inflammatory arm, honokiol reduces hepatic IL-1β through NF-κB/NLRP3 inhibition in liver-injury models [40], whereas the relationship between statins and IL1B is context-dependent rather than uniformly inhibitory. We emphasise that no direct CCL3-expression datum is available for either compound; the CCL3 link rests on docking-based target engagement and broad NF-κB/chemokine suppression, and is therefore presented as a prediction to be tested rather than an established effect. Accordingly, these remain computational and literature-based inferences, and direct experimental confirmation—for example, qPCR and Western-blot measurement of IL1B, CCL3, LUM, SPP1 and ITGA6 in drug-treated hepatic models—is a necessary next step, which we now explicitly flag as a limitation. Furthermore, the molecular docking results presented here represent static binding poses; molecular dynamics (MD) simulations would provide additional insight into the dynamic stability of these protein–ligand interactions and are identified as a priority for future computational validation.

Several limitations warrant consideration. Importantly, the present signature was derived and validated exclusively in liver-tissue transcriptomes and therefore constitutes a tissue-associated rather than a non-invasive biomarker set. Although several signature genes encode secreted or circulating proteins—LUM is an established serum fibrosis marker, and SPP1 (osteopontin), IL1B and CCL3 are detectable in peripheral blood—whether the tissue signature is recapitulated in serum or plasma remains untested. Translating these findings into a non-invasive assay will require paired profiling of tissue and circulating samples, ideally embedded within fibrosis-screening programmes that have been shown to support earlier diagnosis and alcohol abstinence [41]. In addition, the WGCNA co-expression modules and the initial feature-selection step were derived from a small training cohort (*n* = 18; 6 ALC and 12 controls), which may limit module stability and increase the risk of over-fitting. We sought to mitigate this in two ways: the final five-gene signature was retained only at the intersection of three independent algorithms (LASSO, Random Forest and SVM-RFE), and its discriminative performance was confirmed in a fully independent external cohort of 93 samples (AUC = 0.824) rather than being inferred from the training data alone. Nevertheless, formal module-preservation analysis in larger, multi-cohort datasets will be required to establish the robustness of the underlying co-expression structure, and the present modular assignments should be regarded as hypothesis-generating. A further consideration is whether the proposed signature would change with clinical status, particularly following alcohol abstinence. Our cross-sectional, tissue-based design cannot directly address this dynamic behaviour, and we acknowledge it as a limitation. Several lines of evidence nonetheless indicate that the signature has the biological potential to be status-responsive. First, our CTD analysis (Section 2.5) shows that four of the five genes (IL1B, CCL3, SPP1 and ITGA6) are directly ethanol-modulated, indicating that their expression tracks ethanol exposure. Second, sustained abstinence is the single most effective intervention in alcohol-associated liver disease and can drive partial regression of fibrosis, accompanied by reversion of disease-associated epigenetic and transcriptional states toward control [42,43]. Third, however, fibrosis resolution after cessation is non-uniform and can be actively restrained by persistent alcohol-induced epigenetic marks [43], implying that the fibrotic arm of the signature (LUM, SPP1, ITGA6) may decline only partially and variably after drinking stops. Integrating such molecular assessment with fibrosis screening and abstinence counselling, which has measurable clinical benefit [41], could provide a practical setting in which to test signature reversibility. Confirming whether the five-gene signature is itself reversible will require longitudinal sampling of abstinent versus relapsing patients, an important direction for prospective validation. The present diagnostic signature was developed to distinguish ALC tissues from histologically normal liver tissues, rather than to differentiate ALC from cirrhosis of other aetiologies; future studies incorporating multi-aetiology cirrhosis cohorts will be necessary to evaluate the signature’s aetiological specificity. The training cohort comprised a relatively small sample size (*n* = 18), and the retrospective nature precludes assessment of clinical variables such as disease duration and alcohol consumption patterns. While our analysis identified promising candidates, computational predictions require experimental validation in appropriate models before clinical translation. Future studies incorporating single-cell transcriptomics and prospective clinical validation will be essential to translate these findings into improved therapeutic approaches for ALC. Additionally, the immune infiltration analysis relied solely on ssGSEA; future work should employ complementary deconvolution methods such as CIBERSORT or xCell, and ideally single-cell or spatial transcriptomic data, to corroborate the observed macrophage signature attenuation. Finally, the drug repurposing candidates, fluvastatin and honokiol, were identified based on transcriptomic signature reversal in end-stage ALC tissues. Given that their primary diagnostic targets, IL1B and CCL3, are suppressed at this stage owing to CAID-associated immune exhaustion, their direct anti-inflammatory efficacy is most plausibly applicable to earlier, actively inflamed disease stages. This represents an inherent limitation of signature-based repurposing applied to end-stage specimens, and stage-specific experimental validation is warranted.

## 4. Materials and Methods

### 4.1. Study Population and Epidemiological Analysis

Data from 17,007 adults were extracted from the National Health and Nutrition Examination Survey (NHANES, 2017–2023) using the R package nhanesA [44]. Participants with viral hepatitis were excluded. Liver cirrhosis was defined by self-reported physician diagnosis, and alcohol consumption was stratified into never, non-heavy, and heavy drinking (≥4–5 drinks/day). Survey-weighted logistic regression (adjusting for demographics, BMI, and diabetes) was employed to evaluate the epidemiological association between alcohol consumption and cirrhosis risk.

### 4.2. Transcriptomic Data Acquisition and Processing

**Figure 9:**
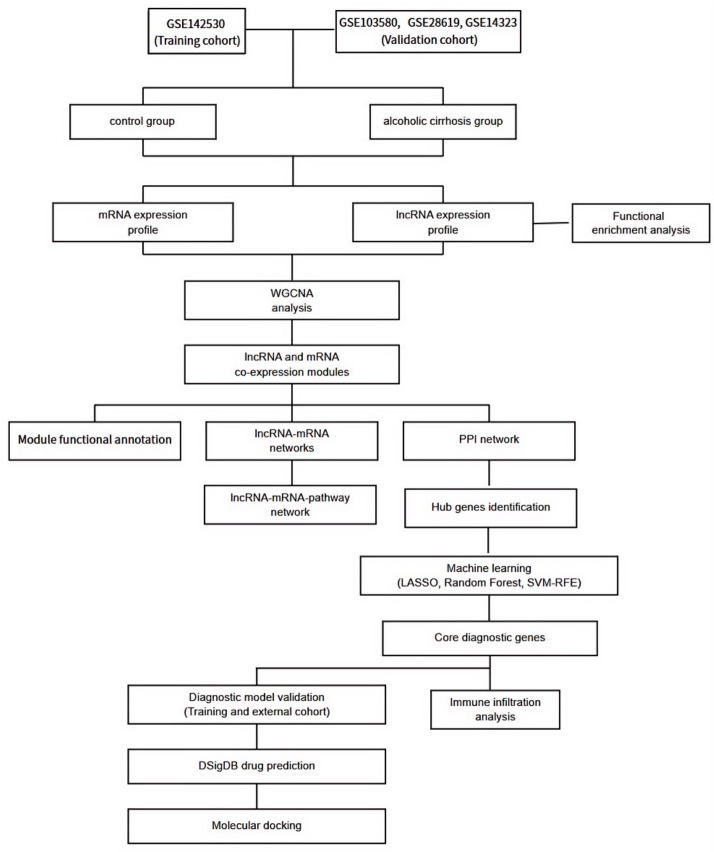
Overview of the study design. The overall study design and analytical workflow, integrating epidemiological data with multi-cohort transcriptomics.

**Figure 10:**
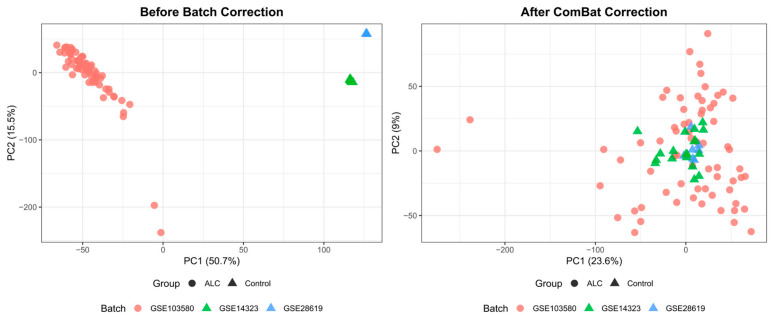
Principal Component Analysis (PCA) of the external validation cohort before and after batch effect correction. (**Left**) PCA plot before correction, showing strong batch-driven clustering. (**Right**) PCA plot after ComBat correction, demonstrating reduced batch effects and improved sample mixing.

The overall study design and analytical workflow, integrating epidemiological data with multi-cohort transcriptomics, are systematically illustrated in Figure 9. Gene expression data for ALC and normal liver tissues were obtained from the Gene Expression Omnibus (GEO) [45]. GSE142530 served as the training cohort (*n* = 18; ALC *n* = 6, normal controls *n* = 12). Three independent datasets (GSE103580, GSE28619, and GSE14323) were merged, rigorously corrected for batch effects using the ComBat function from the sva package (version 3.48.0) [46], and employed as the external validation cohort (*n* = 93). The combined validation cohort consisted of GSE103580 (*n* = 40), GSE28619 (*n* = 22), and GSE14323 (*n* = 31). Each dataset was individually normalised prior to merging using R software (version 4.5.1; R Foundation for Statistical Computing, Vienna, Austria) [47], with raw count data processed using the data.table package (version 1.17.8) [48]. Batch effect correction efficacy was confirmed via Principal Component Analysis (PCA) (Figure 10).

### 4.3. Ethanol–Gene Interaction Validation via the Comparative Toxicogenomics Database

To establish a mechanistic bridge between the population-level epidemiological findings and the transcriptomic signature, the Comparative Toxicogenomics Database (CTD; ctdbase.org) was queried using the chemical term “Ethanol” against each of the five core diagnostic genes (*IL1B*, *CCL3*, *SPP1*, *ITGA6*, and *LUM*). The CTD curates literature-supported chemical–gene interactions, enabling systematic evaluation of whether the identified transcriptomic signature is biologically sensitive to ethanol exposure. Interaction evidence was classified as direct (ethanol shown to alter expression of the target gene or protein in published studies) or indirect (ethanol co-exposure with another chemical yielding the observed transcriptional alterations). This analysis serves to close the logical loop between the epidemiological observation of alcohol-driven cirrhosis risk and the molecular signature derived from ALC transcriptomes.

### 4.4. Co-Expression Network and Machine Learning Feature Selection

Differentially expressed genes (DEGs) were defined using thresholds of adjusted *p* < 0.05 and |log2 fold-change| > 1. Weighted Gene Co-expression Network Analysis (WGCNA) was performed using the WGCNA package [49] to isolate functional gene modules strongly correlated with ALC, with high-variability genes selected based on median absolute deviation (MAD), retaining the top 25% from the variance stabilising transformation (VST)-normalised expression matrix. Gene Ontology (GO) and KEGG pathway enrichment analyses were performed using the clusterProfiler package [50]. Protein–protein interaction (PPI) networks were constructed via the STRING database (minimum high-confidence interaction score threshold of 0.700) [51], and DEGs were imported into Cytoscape software (version 3.10.4) [52] for visualisation. Hub genes were identified using the CytoHubba plugin (Degree method) in Cytoscape [53]. Hub genes from the most significant modules were subjected to three parallel machine learning algorithms—LASSO regression using the glmnet package (version 4.1-7) [54], Random Forest (RF) implemented using the randomForest package (version 4.7-1.1) with 500 trees [55], and SVM-RFE conducted using the e1071 package (version 1.7-13) [56]—to extract a core diagnostic signature. Five-fold cross-validation was used to tune regularisation parameters in LASSO. Given the limited training sample size *n* = 18, the final combined diagnostic model was evaluated using leave-one-out cross-validation (LOOCV) to obtain a bias-corrected estimate of discriminative performance. The diagnostic endpoint was the binary classification of histologically confirmed ALC tissue versus normal liver tissue. Model performance was evaluated via the area under the receiver operating characteristic curve (AUC).

Detailed protocols, including data preprocessing and specific algorithmic configurations, are provided in Supplementary File S1 [57,58].

### 4.5. Immune Infiltration and Therapeutic Prediction

Immune cell abundance, with a specific focus on macrophage populations, was quantified using single-sample Gene Set Enrichment Analysis (ssGSEA) with immune cell gene sets derived from the MSigDB Hallmark and curated immune gene set collections (C7 collection). Immune cell abundance scores were computed per sample and compared between ALC and normal groups using the Wilcoxon rank-sum test. All statistical computations and visualisations were generated using the SciPy (version 1.10.1) [59], Seaborn (version 0.12.2) [60], and Matplotlib (version 3.7.1) [61] libraries in a Python 3.10 environment. Finally, the Drug Signatures Database (DSigDB) [62] was queried using the core gene signature to repurpose candidate therapeutics, which were subsequently validated through structural molecular docking simulations. Molecular docking was performed using AutoDock Vina (version 1.2.3; The Scripps Research Institute, La Jolla, CA, USA) with a grid box centred on the active site of each target protein, for *IL1B* and *CCL3*, the two inflammatory hub genes with available high-resolution crystal structures in the Protein Data Bank; *ITGA6* was excluded from docking analysis due to the absence of a suitable ligand-bound crystal structure. Chemical structures of candidate compounds were obtained from the PubChem database [63]. Ligand geometries were subjected to energy minimisation via ChemBioOffice (PerkinElmer, Waltham, MA, USA) [64]. Receptor structures were retrieved from the RCSB Protein Data Bank [65]. Detailed protocols, including data preprocessing, software versions, and specific algorithmic configurations, are provided in Supplementary File S1.

## 5. Conclusions

This study provides integrated epidemiological and molecular evidence to advance the diagnosis and mechanistic understanding of alcoholic liver cirrhosis. Analysis of 17,007 adults from a nationally representative survey confirmed that heavy drinking independently confers a five-fold increased risk of cirrhosis in a dose-dependent manner. Transcriptomic profiling of human liver tissues revealed pronounced global suppression of long non-coding RNA expression, a finding that may reflect a candidate molecular feature of alcohol-related cirrhosis requiring replication in larger, multi-aetiology cohorts. A five-gene diagnostic signature (*IL1B*, *CCL3*, *LUM*, *SPP1*, *ITGA6*), derived from fibrosis- and immune-associated co-expression modules and developed to distinguish ALC from histologically normal liver tissue, achieved an area under the receiver operating characteristic curve of 0.824 in an independent external validation cohort of 93 samples. Immune microenvironment analysis uncovered global attenuation of macrophage-associated transcriptomic signatures rather than the expected M2-dominant polarisation, suggesting that immunological assessment may inform therapeutic strategy selection at different disease stages. Computational drug repurposing and molecular docking identified fluvastatin and honokiol as candidate therapeutic agents, with fluvastatin showing strong binding affinity to IL1B (−7.65 kcal/mol) and honokiol preferentially targeting CCL3 (−6.94 kcal/mol). Collectively, these findings establish a molecular and epidemiological rationale for developing an ALC-specific diagnostic panel. Because the present signature is tissue-derived, its translation into a non-invasive (blood-based) assay represents an explicit goal for future validation. The analysis also nominates fluvastatin and honokiol as mechanistically plausible therapeutic leads, whose direct anti-inflammatory effects are most pertinent to earlier, actively inflamed stages of alcohol-associated liver disease, and whose anti-fibrotic pleiotropic actions may provide benefit across disease stages—both warranting prospective, stage-stratified clinical validation.

## Figures and Tables

**Table 1:** Baseline characteristics of study participants by liver cirrhosis status, NHANES 2017−2023.

Characteristics	Overall	Non-Cirrhosis	Cirrhosis	*p* Value
Participants, *n*	17,007	16,927	80	
Age, years, mean (SD)	48.60 (17.44)	48.56 (17.44)	58.81 (10.48)	<0.001
**Gender, *n* (%)**				0.318
Male	8393 (48.3)	8349 (48.3)	44 (55.5)	
Female	8614 (51.7)	8578 (51.7)	36 (44.5)	
**Race/Ethnicity, *n* (%)**				0.470
Mexican American	3285 (7.7)	3273 (7.7)	12 (12.3)	
Other Hispanic	2361 (8.6)	2351 (8.6)	10 (9.2)	
Non-Hispanic White	5752 (61.4)	5718 (61.3)	34 (59.1)	
Non-Hispanic Black	3646 (11.3)	3636 (11.3)	10 (5.3)	
Other/Multi-racial	1963 (11.1)	1949 (11.1)	14 (14.0)	
**Education level, *n* (%)**				0.099
Below high school	2916 (10.5)	2898 (10.5)	18 (18.6)	
High school/GED	4008 (26.7)	3986 (26.7)	22 (33.5)	
Some college	4882 (30.0)	4860 (29.9)	22 (33.6)	
College or above	5154 (32.8)	5136 (32.8)	18 (14.3)	
**Family income-to-poverty ratio, *n* (%)**				<0.001
Below poverty	2748 (13.0)	2732 (13.0)	16 (28.4)	
Near poverty	5416 (35.2)	5385 (35.2)	31 (54.0)	
Above poverty	6515 (51.8)	6504 (51.8)	11 (17.6)	
BMI, kg/m^2^, mean (SD)	29.74 (7.32)	29.73 (7.32)	31.03 (7.09)	0.273
**BMI category, *n* (%)**				0.385
<25	4189 (27.0)	4175 (27.1)	14 (16.8)	
25–<30	5142 (31.9)	5120 (31.9)	22 (34.3)	
≥30	5349 (41.1)	5305 (41.1)	44 (48.9)	
Sleep duration, hours, mean (SD)	7.64 (1.52)	7.64 (1.52)	7.72 (2.32)	0.870
**Diabetes status, *n* (%)**				<0.001
No	14,223 (85.7)	14,163 (85.7)	60 (62.9)	
Borderline	553 (2.7)	546 (2.7)	7 (7.9)	
Yes	2216 (11.6)	2203 (11.6)	13 (29.1)	
**Smoking status, *n* (%)**				<0.001
Never/Unknown	9997 (60.0)	9963 (60.0)	34 (30.7)	
Former	4274 (24.4)	4243 (24.4)	31 (49.1)	
Current	2736 (15.6)	2721 (15.6)	15 (20.2)	
**Physical activity, *n* (%)**				0.062
Active	5403 (54.6)	5378 (54.6)	25 (72.7)	
Inactive	4371 (45.4)	4355 (45.4)	16 (27.3)	
**Alcohol drinking status, *n* (%)**				<0.001
Never	1199 (7.7)	1195 (7.7)	4 (5.0)	
Non-heavy	9924 (77.6)	9899 (77.6)	25 (45.2)	
Heavy	2022 (14.7)	1989 (14.7)	33 (49.8)	
**Ever heavy drinker, *n* (%)**				<0.001
No	9937 (84.1)	9899 (84.1)	38 (48.0)	
Yes	2014 (15.9)	1984 (15.9)	30 (52.0)	
Avg drinks/day, median [IQR]	2.00 [1.00, 3.00]	2.00 [1.00, 3.00]	2.05 [2.00, 4.00]	0.007
AST, U/L, median [IQR]	20.00 [16.00, 24.00]	20.00 [16.00, 24.00]	29.11 [22.00, 58.10]	<0.001
ALT, U/L, median [IQR]	18.00 [13.00, 26.00]	18.00 [13.00, 26.00]	25.00 [20.00, 44.01]	<0.001
Platelet count, 10^9^/L, median [IQR]	245.00 [208.00, 288.00]	245.00 [208.00, 288.00]	183.21 [117.21, 250.68]	<0.001
FIB-4 index, median [IQR]	0.86 [0.57, 1.30]	0.86 [0.57, 1.30]	2.27 [1.36, 3.28]	<0.001
**NHANES Cycle, *n* (%)**				0.233
2017–2020	9213 (49.6)	9179 (49.6)	34 (39.3)	
2021–2023	7794 (50.4)	7748 (50.4)	46 (60.7)	

Data are presented as weighted mean (SD) for normally distributed continuous variables, median [IQR] for skewed continuous variables, or unweighted *n* (weighted %) for categorical variables. *p* values were calculated using a survey-weighted *t*-test for continuous variables and the Rao–Scott chi-squared test for categorical variables. Abbreviations: BMI, body mass index; IQR, interquartile range; AST, aspartate aminotransferase; ALT, alanine aminotransferase; FIB-4, Fibrosis-4 index; GED, General Educational Development; NHANES, National Health and Nutrition Examination Survey.

**Table 2:** Survey-weighted logistic regression analysis of the association between alcohol consumption and liver cirrhosis, NHANES 2017–2023.

Exposure	N	Events	Model 1 (Crude)	Model 2	Model 3
**Analysis A: Ever heavy drinker**					
No (reference)	9937	38	1.00	1.00	1.00
Yes	2014	30	5.73 (2.81–11.70) ***	5.48 (2.85–10.60) ***	5.14 (2.60–10.20) ***
*p* for trend			<0.001	<0.001	<0.001
**Analysis B: Drinking status**					
Never (reference)	1199	4	1.00	1.00	1.00
Non-heavy	9924	25	0.89 (0.24–3.30)	1.12 (0.30–4.11)	1.17 (0.32–4.30)
Heavy	2022	33	5.20 (1.53–17.70) *	6.30 (1.91–20.80) **	6.15 (1.87–20.20) **
*p* for trend			<0.001	<0.001	<0.001
**Analysis C: Average drinks per day (continuous)**					
Per 1-drink/day increase	9522	35	1.15 (1.05–1.26) **	1.19 (1.08–1.32) **	1.18 (1.07–1.30) **
*p* for trend			0.005	0.002	0.002

Values are presented as OR (95% CI). All analyses incorporated NHANES survey weights to account for the complex sampling design. Model 1: Unadjusted. Model 2: Adjusted for age, gender, and race/ethnicity. Model 3: Further adjusted for education, BMI, and diabetes status. * *p* < 0.05; ** *p* < 0.01; *** *p* < 0.001. Heavy drinking was defined based on NHANES questionnaire ALQ151 (“Ever have 4/5 or more drinks every day?”). Abbreviations: OR, odds ratio; CI, confidence interval; BMI, body mass index; NHANES, National Health and Nutrition Examination Survey.

## Data Availability

The transcriptomic datasets analysed in this study are publicly available from the Gene Expression Omnibus (GEO) repository under the following accession numbers: GSE142530, GSE103580, GSE28619, and GSE14323. The epidemiological data are publicly available from the National Health and Nutrition Examination Survey (NHANES) at https://www.cdc.gov/nchs/nhanes/ (accessed on 3 January 2026). All other data supporting the findings of this study are available within the article and its Supplementary Materials.

## Supplementary Materials

The following supporting information can be downloaded at: https://www.mdpi.com/article/10.3390/ijms27135809/s1.

## Abbreviations

The following abbreviations are used in this manuscript:

ALC	Alcohol-associated liver cirrhosis
NHANES	National Health and Nutrition Examination Survey
GEO	Gene Expression Omnibus
DEG	Differentially expressed gene
lncRNA	Long non-coding RNA
mRNA	Messenger RNA
WGCNA	Weighted gene co-expression network analysis
LASSO	Least absolute shrinkage and selection operator
SVM-RFE	Support vector machine–recursive feature elimination
RF	Random Forest
AUC	Area under the receiver operating characteristic curve
LOOCV	Leave-one-out cross-validation
PPI	Protein–protein interaction
ssGSEA	Single-sample gene set enrichment analysis
ECM	Extracellular matrix
CTD	Comparative Toxicogenomics Database
DSigDB	Drug Signatures Database
MASLD	Metabolic dysfunction-associated steatotic liver disease
CAID	Cirrhosis-associated immune dysfunction
HCC	Hepatocellular carcinoma
PCA	Principal Component Analysis
BMI	Body mass index
OR	Odds ratio
CI	Confidence interval
FIB-4	Fibrosis-4 index
AST	Aspartate aminotransferase
ALT	Alanine aminotransferase
IQR	Interquartile range
TGF-β	Transforming growth factor-beta
EMT	Epithelial-mesenchymal transition
DNMT	DNA methyltransferase
HDAC	Histone deacetylase
