# Supplementary material for: Transcriptomic Identification of Diagnostic Biomarkers for Alcohol-Associated Liver Cirrhosis: Integration of Population-Level Epidemiology with Multi-Cohort Transcriptomic Analysis
Source: Int J Mol Sci. 2026 Jun 26;27(13):5809. doi: 10.3390/ijms27135809 (PMC13360815; doi:10.3390/ijms27135809)
Supplement: Supplementary file 1 [file ijms-27-05809-s001.zip › ijms-4362915 - Supplementary_File_1.pdf]

International Journal of
Molecular Sciences

MDPI

Article

Transcriptomic Identification of Diagnostic Biomarkers for Alcohol-Associated Liver Cirrhosis: Integration of Population-Level Epidemiology with Multi-Cohort Transcriptomic Analysis

Hao Wang [1], Wenzhang Ding [2], Linjie Zhang [3], Muyang Xu [1] and Jing Sui [3,4,*]

Supplementary File S1: Detailed Materials and Methods

1. Population-Based Epidemiological Analysis

Data from the NHANES (2017–2023) were extracted using the R package nhanesA. The NHANES utilises a complex, stratified, multistage probability cluster design [44]. Alcohol consumption was classified into never, non-heavy, and heavy drinking (≥4–5 drinks/day, ALQ151), with continuous daily intake also assessed [57]. Statistical analyses accounted for the complex survey design by incorporating primary sampling units (SDMVPSU), stratification (SDMVSTRA), and recalculated 4-year examination weights to ensure national representativeness [58].

2. Data Preprocessing and Batch Effect Removal

Gene expression datasets related to ALC were retrieved from the Gene Expression Omnibus (GEO) database [45]. All statistical analyses and data processing were performed using R software (version 4.5.1) [47]. Raw count data from GSE142530 were processed using the data.table package (version 1.17.8) [48]. For the validation cohort (GSE103580, GSE28619, and GSE14323), batch effects across different sequencing platforms were corrected using the ComBat function from the sva package (version 3.48.0)[46].

3. Weighted Gene Co-expression Network Analysis (WGCNA)

The co-expression network was constructed using the WGCNA package [49]. High-variability genes were selected based on MAD, retaining the top 25% from the variance stabilizing transformation (VST)-normalised expression matrix.

4. Functional Enrichment and PPI Network

GO and KEGG pathway enrichment analyses were performed using the clusterProfiler package [50]. PPI networks were constructed via the STRING database using a minimum high-confidence interaction score threshold of 0.700 [51]. DEGs were imported into Cytoscape software for visualization [52]. Hub genes were identified using the CytoHubba plugin (Degree method) in Cytoscape [53].

5. Machine Learning Feature Selection

LASSO regression: Performed using the glmnet package (version 4.1-7) [54].

Random Forest (RF): Implemented using the randomForest package (version 4.7-1.1) with 500 trees [55].

 https://doi.org/10.3390/ijms27135809

SVM-RFE: Conducted using the e1071 package (version 1.7-13) [56].

6. Immune Infiltration Assessment

Immune cell infiltration was assessed via the ssGSEA algorithm, using immune cell-type gene sets derived from the MSigDB Hallmark collection and curated immunological signatures (C7 collection). Immune cell abundance scores were computed per sample and compared between ALC and normal groups using the Wilcoxon rank-sum test. All statistical computations and visualizations were generated using the SciPy (version 1.10.1) [59], Seaborn (version 0.12.2) [60], and Matplotlib (version 3.7.1) [61] libraries in a Python 3.10 environment.

7. Drug Repurposing and Molecular Docking

Drug repurposing was performed using the DSigDB [62]. Molecular docking was conducted using AutoDock Vina (version 1.2.3) with a grid box centred on the active site of each target protein. Chemical structures of Fluvastatin and Honokiol were obtained from the PubChem database [63]. Ligand geometries were subjected to energy minimization via ChemBioOffice [64]. Receptor structures were retrieved from the RCSB Protein Data Bank [65].

Supplementary References

Supplementary references have also been cited in the paper. The numbering of the supplementary references in this file corresponds to the numbering in the paper.

44. Choday, S.; Zahdeh, T.; Kang, P.; et al. Insights on Alcohol-Associated Liver Disease, a Decade of Data from National Survey. *Gastrointest. Disord.* **2025**, *7*, 52.
45. Clough, E.; Barrett, T.; Wilhite, S.E.; et al. NCBI GEO: Archive for gene expression and epigenomics data sets: 23-year update. *Nucleic Acids Res.* **2024**, *52*, D138–D144.
46. Leek, J.T.; Johnson, W.E.; Parker, H.S.; et al. The sva package for removing batch effects and other unwanted variation in high-throughput experiments. *Bioinformatics* **2012**, *28*, 882–883.
47. R Core Team. R: A Language and Environment for Statistical Computing; R Foundation for Statistical Computing: Vienna, Austria, 2025.
48. Dowle, M.; Srinivasan, A. data.table: Extension of data.frame, R Package Version 1.17.8; 2024.
49. Langfelder, P.; Horvath, S. WGCNA: An R package for weighted correlation network analysis. *BMC Bioinformatics* **2008**, *9*, 559.
50. Wu, T.; Hu, E.; Xu, S.; et al. clusterProfiler 4.0: A universal enrichment tool for interpreting omics data. *Innovation* **2021**, *2*, 100141.
51. Szklarczyk, D.; Nastou, K.; Koutrouli, M.; Kirsch, R.; Mehryary, F.; Hachilif, R.; Hu, D.; Peluso, M.E.; Huang, Q.; Fang, T.; Doncheva, N.T. et al. The STRING database in 2025: Protein networks with directionality of regulation. *Nucleic Acids Res.* **2025**, *53*, D730–D737.
52. Shannon, P.; Markiel, A.; Ozier, O.; Beliga, N.S.; Eang, J.T.; Ramage, D.; Amin, N.; Schwikowiski B.; Ideker. T. Cytoscape: A software environment for integrated models of biomolecular interaction networks. *Genome Res.* **2003**, *13*, 2498–2504.
53. Chin, C.H.; Chen, S.H.; Wu, H.H.; Ho, C.-W.; Ko, M.-T.; Lin, C.-Y. cytoHubba: Identifying hub objects and sub-networks from complex interactome. *BMC Syst. Biol.* **2014**, *8*, S11.
54. Tay, J.K.; Narasimhan, B.; Hastie, T. Elastic net regularization paths for all generalized linear models. *J. Stat. Softw.* **2023**, *106*, 1–31.
55. Breiman, L. Random forests. *Mach. Learn.* **2001**, *45*, 5–32.
56. Meyer, D.; Dimitriadou, E.; Hornik, K.; Weingessel, A.; Leisch, F. e1071: Misc Functions of the Department of Statistics, Probability Theory Group (Formerly: E1071), TU Wien, R Package Version 1.7-13; 2019.
57. Ma, C.; Zhang, X.; Zhang, W.; Duan, J.; Yang, H. Association between serum homocysteine levels and advanced hepatic fibrosis in alcohol-related liver disease: A cross-sectional study of NHANES. *Medicine* **2025**, *104*, e43395.
58. Li, X.M.; Liu, S.L.; He, Y.J.; Shu, J.C. Using new indices to predict metabolism dysfunction-associated fatty liver disease (MAFLD): Analysis of the National Health and Nutrition Examination Survey database. *BMC Gastroenterol.* **2024**, *24*, 109.

https://doi.org/10.3390/ijms27135809

59. Virtanen, P.; Gommers, R.; Oliphant, T.E.; Haberland, M.; Reddy, T.; Cournapeau, D.; Burovski, E.; Peterson, P.; Weckesser, W.; Bright, J. SciPy 1.0: Fundamental algorithms for scientific computing in Python. *Nat. Methods* **2020**, *17*, 261–272.
60. Waskom, M.L. seaborn: Statistical data visualization. *J. Open Source Softw.* **2021**, *6*, 3021.
61. Hunter, J.D. Matplotlib: A 2D graphics environment. *Comput. Sci. Eng.* **2007**, *9*, 90–95.
62. Yoo, M.; Shin, J.; Kim, J.; Kim, J.; Ryall, K.A.; Lee, K.; Lee, S.; Jeon, M.; Kang, J.; Tan, A.C. DSigDB: Drug signatures database for gene set analysis. *Bioinformatics* **2015**, *31*, 3069–3071.
63. Kim, S.; Chen, J.; Cheng, T.; Gindulyte, A.; He, J.; He, S.; Li, Q.; Scoemaker, B.A.; Thiessen, P.A.; Yu, B. et al. PubChem 2025 update. *Nucleic Acids Res.* **2025**, *53*, D1516–D1525.
64. Kerwin, S.M. ChemBioOffice Ultra 2010 suite. *J. Am. Chem. Soc.* **2010**, *132*, 2466–2467.
65. Burley, S.K.; Bhikadiya, C.; Bi, C.; Bittrich, S.; Chao, H.; Craig, P.A.; Crichlow, G.V.; Dalenberg, K.; Duarte, J.M.; Dutta, S. et al. RCSB Protein Data Bank (RCSB.org): Delivery of experimentally-determined PDB structures alongside one million computed structure models of proteins from artificial intelligence/machine learning. *Nucleic Acids Res.* **2023**, *51*, D488–D508.

https://doi.org/10.3390/ijms27135809
