# Supplementary material for: Transcriptomic Identification of Diagnostic Biomarkers for Alcohol-Associated Liver Cirrhosis: Integration of Population-Level Epidemiology with Multi-Cohort Transcriptomic Analysis
Source: Int J Mol Sci. 2026 Jun 26;27(13):5809. doi: 10.3390/ijms27135809 (PMC13360815; doi:10.3390/ijms27135809)
Supplement: Supplementary file 1 [file ijms-27-05809-s001.zip › ijms-4362915 - Supplementary_Table.pdf]

International Journal of
Molecular Sciences

MDPI

Article

Transcriptomic Identification of Diagnostic Biomarkers for Alcohol-Associated Liver Cirrhosis: Integration of Population-Level Epidemiology with Multi-Cohort Transcriptomic Analysis

Hao Wang [1], Wenzhang Ding [2], Linjie Zhang [3], Muyang Xu [1] and Jing Sui [3,4,*]

Table S1. The distribution of mRNAs, lncRNAs across the identified co-expression modules.

Module	Total Genes	mRNAs	lncRNAs
Grey	2430	1,957	473
Turquoise	1415	831	584
Brown	1141	997	144
Yellow	877	789	88
Red	298	222	76
Black	233	130	103
Pink	174	152	22
Purple	99	43	56
Tan	70	66	4

Table S2. Predicted therapeutic drugs for ALC.

Compound	P value	Odds Ratio	Combined Score	Predicted Target Genes
Fluvastatin PC3 UP	1.50E-05	170.658	578.898	IL1B; ITGA6
Honokiol CTD 00000234	2.02E-05	102.333	493.037	IL1B; CCL3
