# Supplementary material for: Transcriptomic Identification of Diagnostic Biomarkers for Alcohol-Associated Liver Cirrhosis: Integration of Population-Level Epidemiology with Multi-Cohort Transcriptomic Analysis
Source: Int J Mol Sci. 2026 Jun 26;27(13):5809. doi: 10.3390/ijms27135809 (PMC13360815; doi:10.3390/ijms27135809)
Supplement: Supplementary file 1 [file ijms-27-05809-s001.zip › ijms-4362915 Supplementary Figure.pdf]

International Journal of
Molecular Sciences

Article

Transcriptomic Identification of Diagnostic Biomarkers for Alcohol-Associated Liver Cirrhosis: Integration of Population-Level Epidemiology with Multi-Cohort Transcriptomic Analysis

Hao Wang [1], Wenzhang Ding [2], Linjie Zhang [3], Muyang Xu [1] and Jing Sui [3,4,*]

 https://doi.org/10.3390/ijms27135809

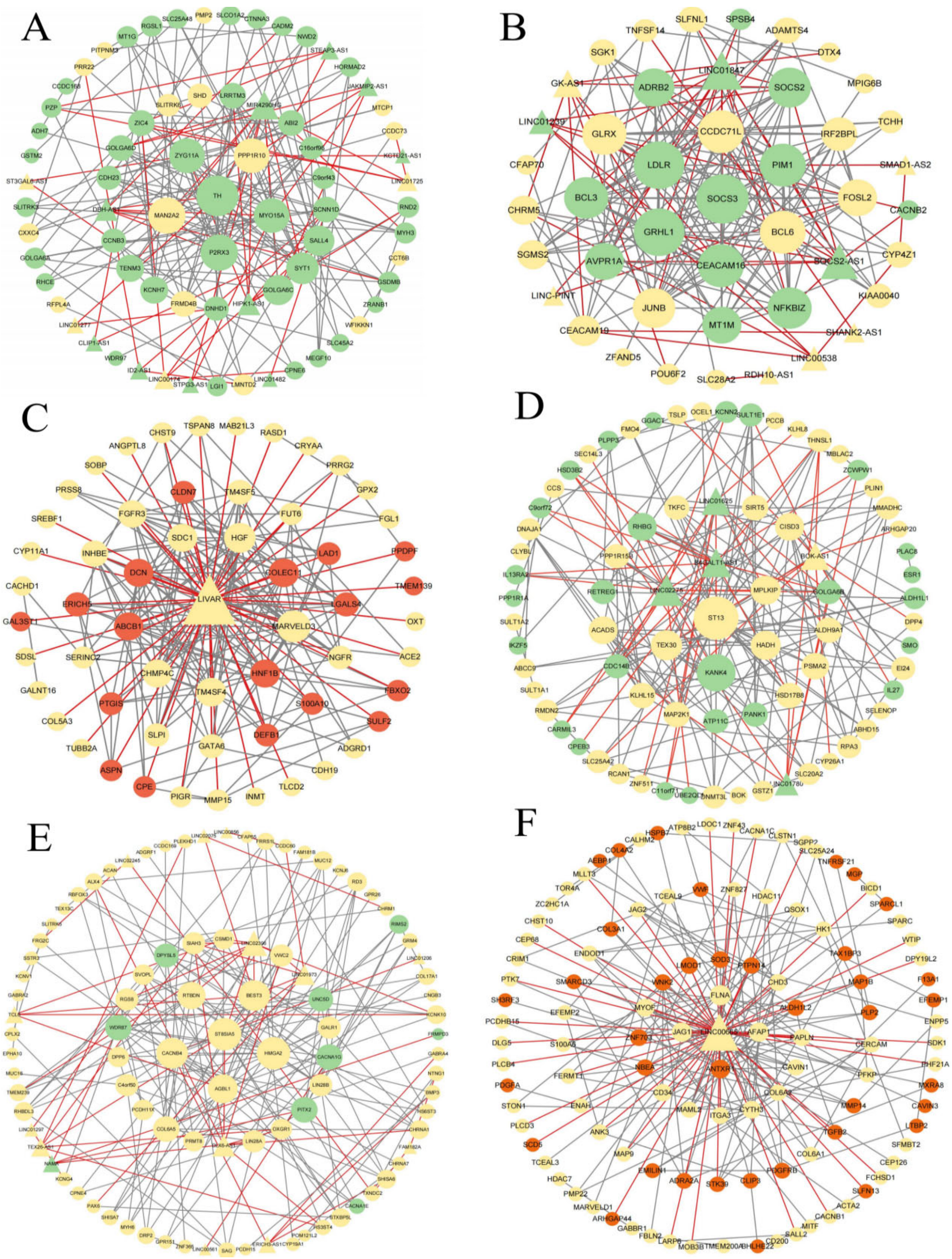

Supplementary Figure S1. LncRNA-mRNA co-expression networks of the remaining six modules identified by WGCNA. The co-expression patterns were visualized for the (A) Black, (B) Purple, (C) Tan, (D) Red, (E) Turquoise, and (F) Brown modules. Node attributes: Triangles represent lncRNAs, and circles represent mRNAs. Color codes: Red nodes indicate upregulated genes, Green nodes indicate downregulated genes, and Yellow nodes indicate genes with no significant differential expression (NS) or intermediate regulation. Edge attributes: Red lines represent co-expression interactions between lncRNA and mRNA pairs.

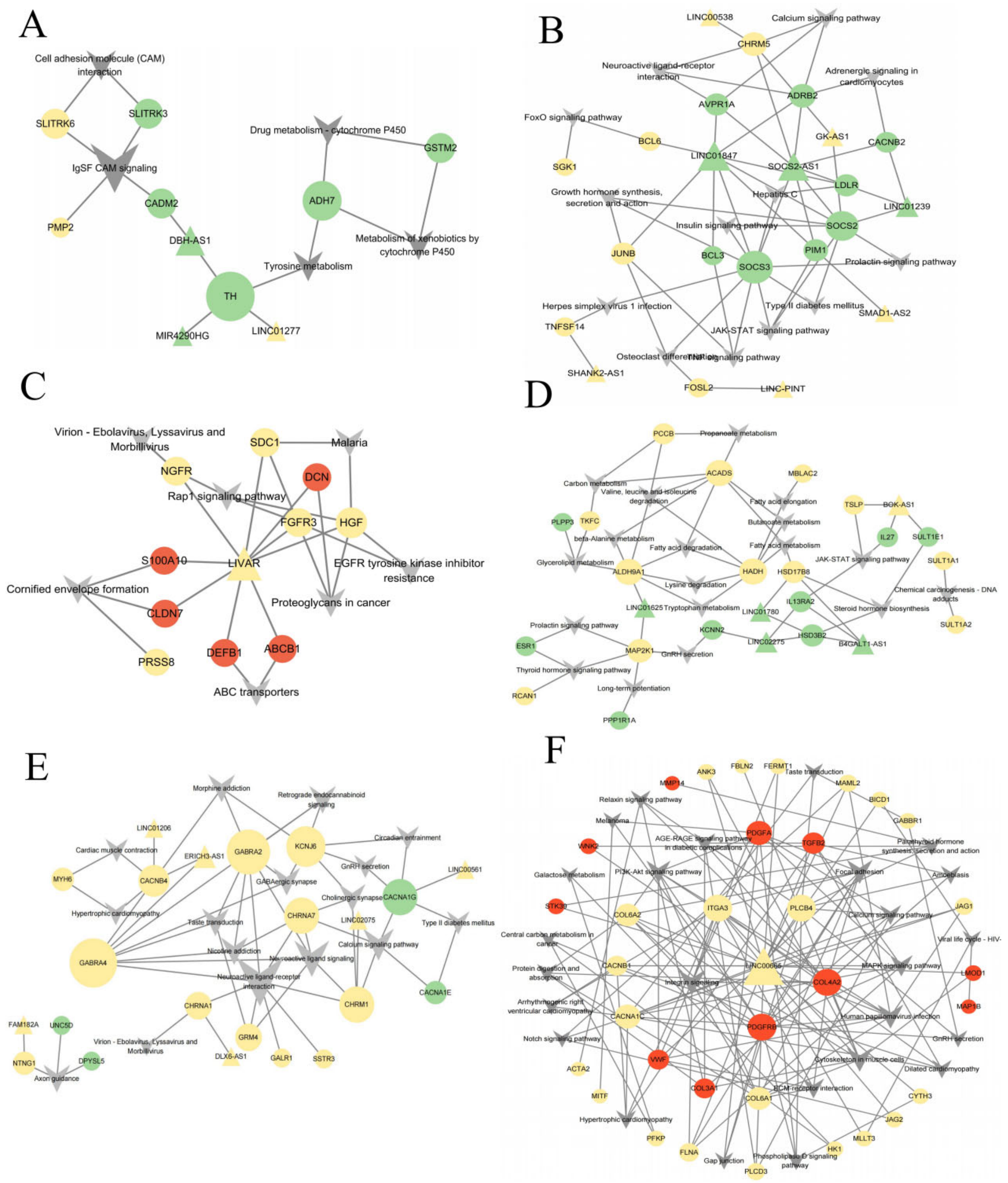

Supplementary Figure S2. LncRNA-mRNA-pathway interaction networks of the remaining six modules.The networks illustrate the regulatory relationships between lncRNAs, target mRNAs, and enriched KEGG pathways for the (A) Black, (B) Purple, (C) Tan, (D) Red, (E) Turquoise, and (F) Brown modules. (A) Black module: Characterized by the downregulation of metabolic genes (ADH7, GSTM2) and pathways related to Drug metabolism - cytochrome P450 and Tyrosine metabolism. (B) Purple module: Centered on SOCS3 and SOCS2, involving JAK-STAT signaling and Insulin signaling pathways, suggesting a role in cytokine regulation. (C) Tan module: Features upregulation of HGF and FGFR3, enriched in Proteoglycans in cancer and Rap1 signaling, indicating regenerative or tumorigenic potential. (D) Red module: Associated with metabolic impairment, specifically Fatty acid degradation and Valine, leucine and isoleucine degradation. (E) Turquoise module: Involves Calcium signaling and Neuroactive ligand-receptor interaction, reflecting alterations in ion channels and neurotransmission. (F) Brown module: Represents a secondary fibrotic network enriched in Focal adhesion, PI3K-Akt signaling, and ECM-receptor interaction, driven by profibrogenic markers such as TGFB2 and PDGFA. Node attributes: V-shapes represent KEGG pathways, triangles represent lncRNAs, and circles represent mRNAs.

https://doi.org/10.3390/ijms27135809

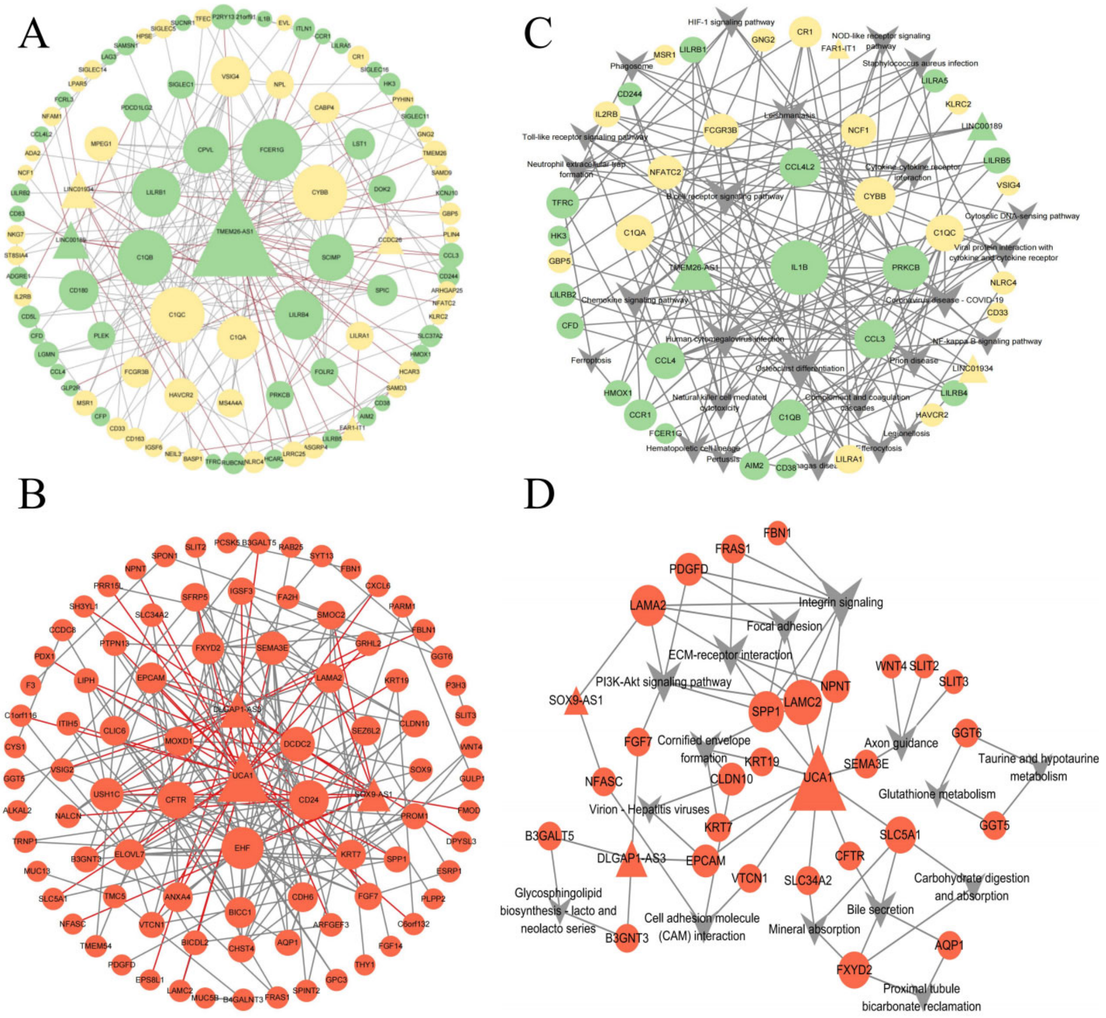

Supplementary Figure S3. Construction of lncRNA-mRNA and pathway interaction networks. (A-B) The lncRNA-mRNA co-expression networks for the pink module (A) and the yellow module (B). (C-D) The lncRNA-mRNA-pathway interaction networks for the pink module (C) and the yellow module (D). Node attributes: Triangles represent lncRNAs, circles represent mRNAs, and V-shapes represent pathways. Edge attributes: Red lines represent interactions between lncRNAs and mRNAs. colour codes: Red nodes indicate upregulated expression, green nodes indicate downregulated expression, and yellow nodes indicate pathways or nodes with mixed regulation (NS).

https://doi.org/10.3390/ijms27135809

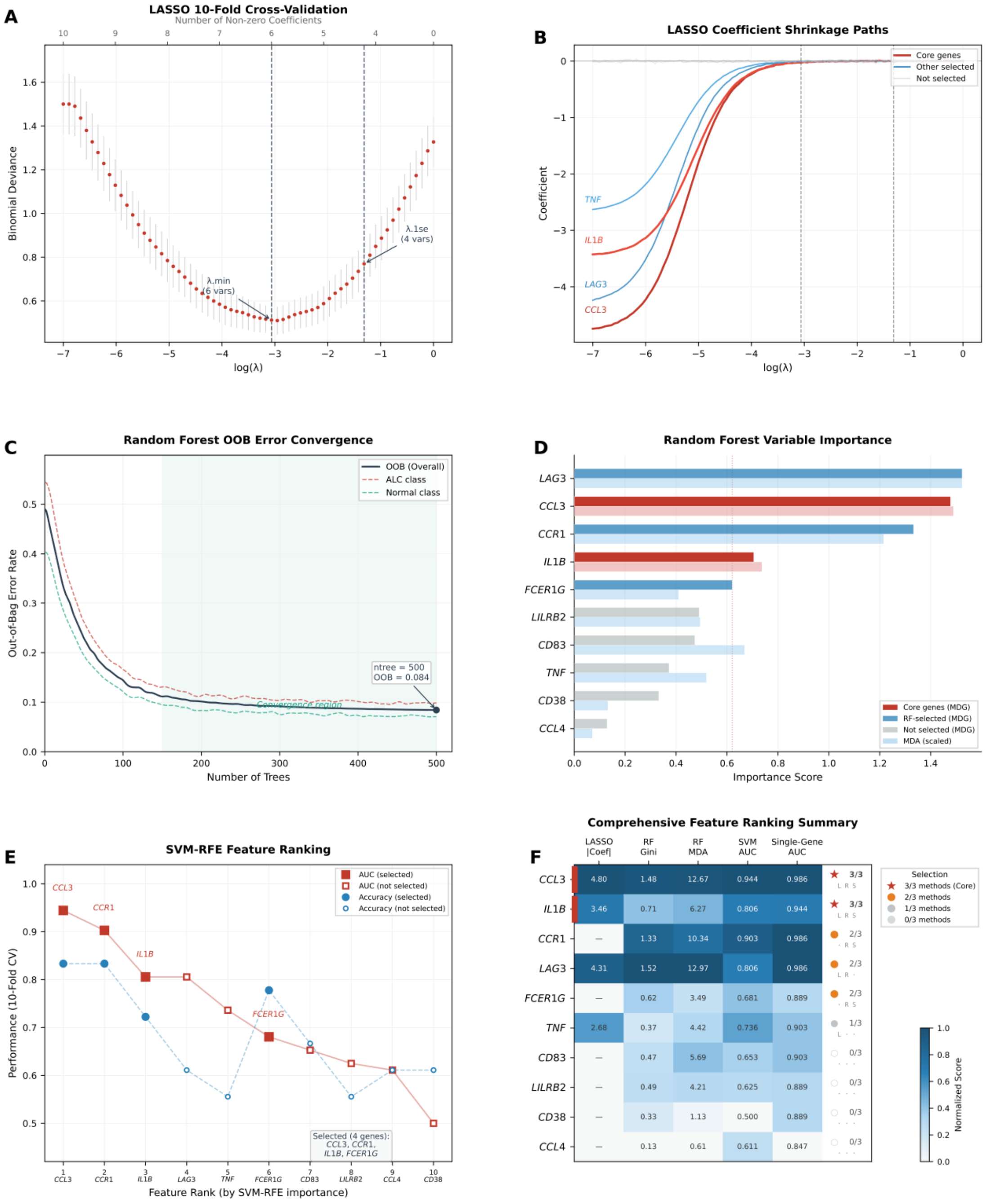

Supplementary Figure S4. Machine learning-based feature selection for the Pink module. (A) LASSO 10-fold cross-validation plot showing binomial deviance as a function of log(λ). Vertical dashed lines indicate λ.min (6 variables) and λ.1se (4 variables); the λ.1se model was selected for parsimony. (B) LASSO coefficient shrinkage paths for hub genes. Core genes (CCL3, IL1B) are highlighted in red; the vertical dashed line indicates the selected λ.1se. (C) Random Forest out-of-bag (OOB) error convergence plot across 500 trees. The overall OOB error rate stabilized at 0.084 (convergence at approximately 160 trees). Dashed lines represent class-specific error rates for ALC (red) and Normal (green). (D) Random Forest variable importance ranked by Mean Decrease in Gini (MDG, bars) and Mean Decrease in Accuracy (MDA, scaled; light overlay). The top 5 genes (LAG3, CCL3, CCR1, IL1B, FCER1G) were selected as RF-identified features. Core genes are shown in red. (E) SVM-RFE feature ranking plot displaying 10-fold cross-validated AUC (red) and accuracy (blue) as features are sequentially eliminated. The optimal subset comprised 4 features (CCL3, CCR1, IL1B, FCER1G). (F) Comprehensive feature ranking summary integrating results from all three methods. The heatmap displays normalized scores for LASSO coefficients, RF Gini importance, RF MDA, SVM AUC, and single-gene AUC. Selection consensus is annotated as 3/3 (core gene, red star), 2/3

(orange circle), 1/3 (yellow circle), or 0/3 (gray circle). L, R, and S denote selection by LASSO, Random Forest, and SVM-RFE, respectively. The intersection of all three methods identified CCL3 and IL1B as core diagnostic genes.

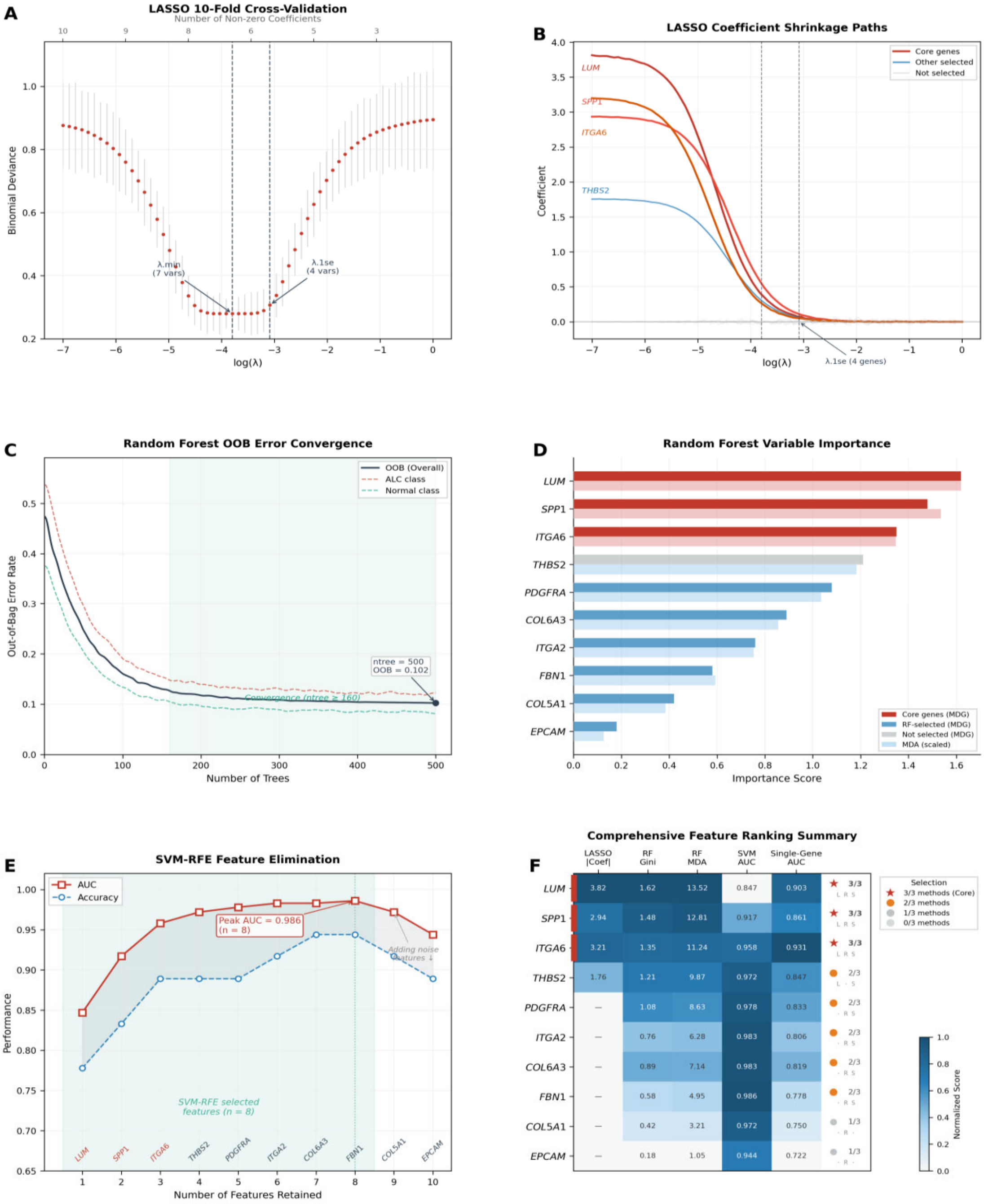

Supplementary Figure S5. Machine learning-based feature selection for the Yellow module. (A) LASSO 10-fold cross-validation plot showing binomial deviance as a function of log(λ). Vertical dashed lines indicate λ.min (7 variables) and λ.1se (4 variables); the λ.1se model was selected for parsimony. (B) LASSO coefficient shrinkage paths for hub genes. Core genes (LUM, SPP1, ITGA6) are highlighted in red; the vertical dashed line indicates the selected λ.1se. THBS2 (blue) was retained at λ.1se but was not confirmed by all three methods. (C) Random Forest OOB error convergence plot across 500 trees. The overall OOB error rate stabilized at 0.102 (convergence at approximately 160 trees). Dashed lines represent class-specific error rates for ALC (red) and Normal (green). (D) Random Forest variable importance ranked by Mean Decrease in Gini (MDG, bars) and Mean Decrease in Accuracy (MDA, scaled; light overlay). The top 9 genes were selected as RF-identified features based on importance scores. Core genes are shown in red. (E) SVM-RFE feature elimination

https://doi.org/10.3390/ijms27135809

plot displaying AUC (red) and accuracy (blue) across sequential feature elimination steps. Peak AUC of 0.986 was achieved with 8 features; performance declined upon inclusion of additional features (COL5A1, EPCAM), suggesting introduction of noise. (F) Comprehensive feature ranking summary integrating results from all three methods. The heatmap displays normalized scores for LASSO coefficients, RF Gini importance, RF MDA, SVM AUC, and single-gene AUC. Selection consensus is annotated as 3/3 (core gene, red star), 2/3 (orange circle), 1/3 (yellow circle), or 0/3 (gray circle). L, R, and S denote selection by LASSO, Random Forest, and SVM-RFE, respectively. The intersection of all three methods identified LUM, SPP1, and ITGA6 as core diagnostic genes.

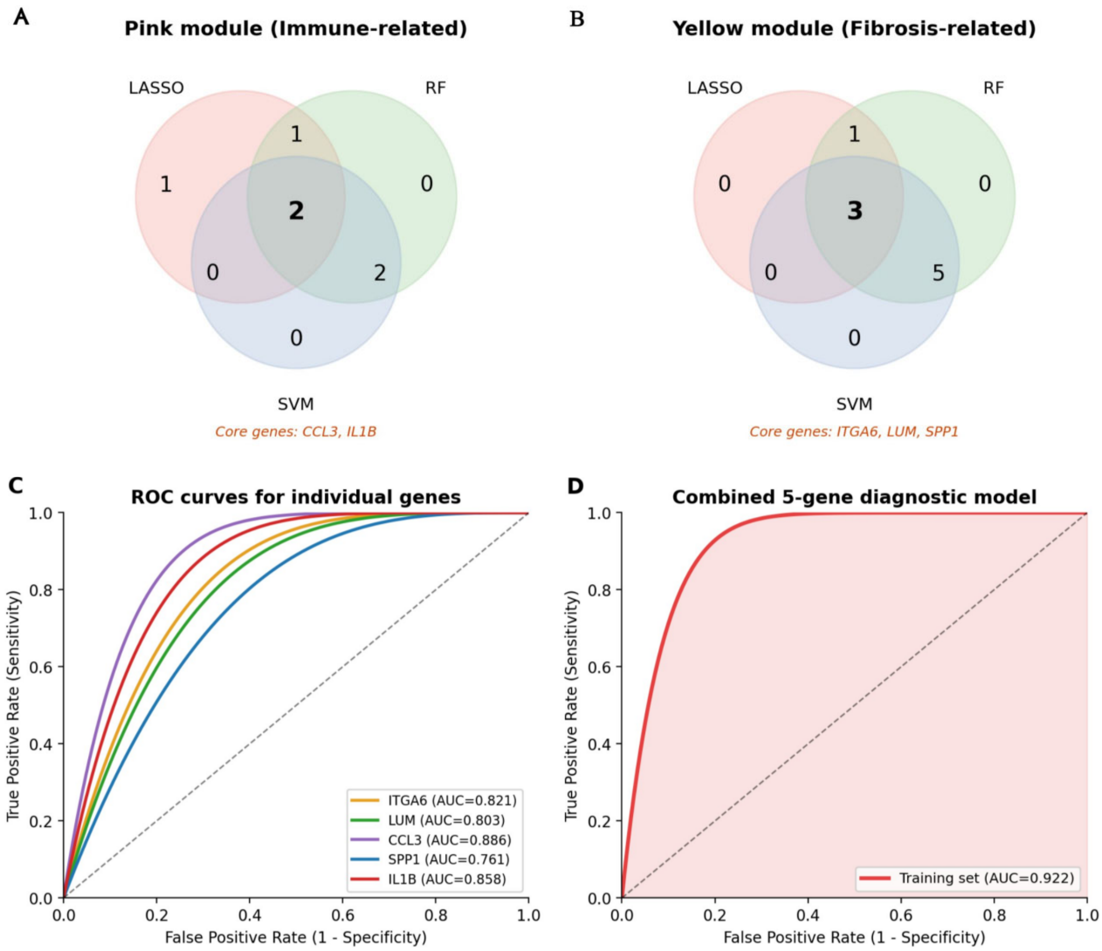

Supplementary Figure S6. Machine learning feature selection and diagnostic model validation. (A-B) Venn diagrams showing the intersection of LASSO, RF, and SVM-RFE in the pink module (A) and yellow module (B). (C) ROC curves of individual core genes in the training set. (D) ROC curve of the combined 5-gene diagnostic model in the training cohort (AUC=0.922).
